# Vascular Endothelial Growth Factor Mediates Intracrine Survival in Human Breast Carcinoma Cells through Internally Expressed VEGFR1/FLT1

**DOI:** 10.1371/journal.pmed.0040186

**Published:** 2007-06-05

**Authors:** Tae-Hee Lee, Seyha Seng, Masayuki Sekine, Cimona Hinton, Yigong Fu, Hava Karsenty Avraham, Shalom Avraham

**Affiliations:** Division of Experimental Medicine, Beth Israel Deaconess Medical Center and Harvard Medical School, Boston, Massachusetts, United States of America; Sunnybrook and Women's College Health Sciences Centre, Canada

## Abstract

**Background:**

While vascular endothelial growth factor (VEGF) expression in breast tumors has been correlated with a poor outcome in the pathogenesis of breast cancer, the expression, localization, and function of VEGF receptors VEGFR1 (also known as FLT1) and VEGFR2 (also known as KDR or FLK1), as well as neuropilin 1 (NRP1), in breast cancer are controversial.

**Methods and Findings:**

We investigated the expression and function of VEGF and VEGF receptors in breast cancer cells. We observed that VEGFR1 expression was abundant, VEGFR2 expression was low, and NRP1 expression was variable. MDA-MB-231 and MCF-7 breast cancer cells, transfected with antisense *VEGF* cDNA or with siVEGF (*VEGF*-targeted small interfering RNA), showed a significant reduction in VEGF expression and increased apoptosis as compared to the control cells. Additionally, specifically targeted knockdown of VEGFR1 expression by siRNA (siVEGFR1) significantly decreased the survival of breast cancer cells through down-regulation of protein kinase B (AKT) phosphorylation, while targeted knockdown of VEGFR2 or NRP1 expression had no effect on the survival of these cancer cells. Since a VEGFR1-specific ligand, placenta growth factor (PGF), did not, as expected, inhibit the breast cancer cell apoptosis induced by siVEGF, and since VEGFR1 antibody also had no effects on the survival of these cells, we examined VEGFR1 localization. VEGFR1 was predominantly expressed internally in MDA-MB-231 and MCF-7 breast cancer cells. Specifically, VEGFR1 was found to be colocalized with lamin A/C and was expressed mainly in the nuclear envelope in breast cancer cell lines and primary breast cancer tumors. Breast cancer cells treated with siVEGFR1 showed significantly decreased VEGFR1 expression levels and a lack of VEGFR1 expression in the nuclear envelope.

**Conclusions:**

This study provides, to our knowledge for the first time, evidence of a unique survival system in breast cancer cells by which VEGF can act as an internal autocrine (intracrine) survival factor through its binding to VEGFR1. These results may lead to an improved strategy for tumor therapy based on the inhibition of angiogenesis.

## Abstract 2

Shalom Avraham and colleagues' study provides evidence of a survival system in breast cancer cells by which VEGF acts as an internal autocrine survival factor through its binding to VEGFR1.

## Editors' Summary

**Background.:**

One woman in eight will develop breast cancer during her lifetime. Most of these women live for many years after their diagnosis and many are cured of their cancer. However, sometimes the cancer grows inexorably and spreads (metastasizes) around the body despite the efforts of oncologists. Characteristics of the tumor known as prognostic factors can indicate whether this spreading is likely to happen. Large tumors that have metastasized have a poorer prognosis than small tumors that are confined to the breast. The expression of specific proteins within the tumor also provides prognostic information. One protein whose expression is associated with a poor prognosis is vascular endothelial growth factor (VEGF). VEGF stimulates angiogenesis—the growth of new blood vessels. Small tumors get the nutrients needed for their growth from existing blood vessels but large tumors need to organize their own blood supply. They do this, in part, by secreting VEGF. This compound binds to proteins (receptors) on the surface of endothelial cells (the cells lining blood vessels), which then send a signal into the cell instructing it to make new blood vessels. Angiogenesis inhibitors, including molecules that block the activity of VEGF receptors, are being developed for the treatment of cancer.

**Why Was This Study Done?:**

Some breast cancer cell lines (cells isolated from breast cancers and grown in the laboratory) make VEGF and VEGF receptors (VEGFR1, VEGFR2, and neuropilin 1 [NRP1]). But, although some studies have reported an association between VEGFR1 expression in breast tumors and a poor prognosis, other studies have found no expression of VEGFR1 in breast tumors. Consequently, the role of VEGF receptors in breast cancer is unclear. In this study, the researchers analyzed the expression and function of VEGF and its receptors in breast cancer cells to investigate whether and how VEGF helps these cells to survive.

**What Did the Researchers Do and Find?:**

The researchers first examined the expression of VEGF receptors in several human breast cancer cell lines. All of them expressed VEGFR1, some expressed NRP1, but VEGFR2 expression was universally low. They then investigated the function of VEGF and its receptors in two human breast cancer cell lines (MDA-MB-231 and MCF-7). In both cell lines, blocking the expression of VEGF or of VEGFR1 (but not of the other two receptors) reduced cell survival by stimulating a specific process of cell death called apoptosis. Unexpectedly, adding VEGF to the cultures did not reverse the effect of blocking VEGF expression, a result that suggests that VEGF and VEGFR1 do not affect breast cancer cell survival by acting at the cell surface. Accordingly, when the researchers examined where VEGFR1 occurs in the cell, they found it on the membranes around the nucleus of the breast cancer cell lines and not on the cell surface; several primary breast tumors and normal breast tissue had the same localization pattern. Finally, the researchers showed that inhibitors of VEGF action that act at the cell surface did not affect the survival of the breast cancer cell lines.

**What Do These Findings Mean?:**

These findings suggest that VEGF helps breast cancer cells to survive in a unique way: by binding to VEGFR1 inside the cell. In other words, whereas VEGF normally acts as a paracrine growth factor (it is released by one cell and affects another cell), in breast cancer cells it might act as an internal autocrine (intracrine) survival factor, a factor that affects the cells in which it is produced. These findings need confirming in more cell lines and in primary breast cancers but could have important implications for the treatment of breast cancer. Inhibitors of VEGF and VEGFR1 that act inside the cell (small molecule drugs) might block breast cancer growth more effectively than inhibitors that act at the cell surface (for example, proteins that bind to the receptor), because internally acting inhibitors might both kill the tumor directly and have antiangiogenic effects, whereas externally acting inhibitors could only have the second effect.

**Additional Information.:**

Please access these Web sites via the online version of this summary at http://dx.doi.org/10.1371/journal.pmed.0040186.

- US National Cancer Institute information for patients and professionals on breast cancer (in English and Spanish) and on angiogenesis (in English and Spanish)
- MedlinePlus Encyclopedia information for patients on breast cancer (in English and Spanish)
- CancerQuest, information from Emory University on cancer biology and on angiogenesis and angiogenesis inhibitors  (in several languages)
- Wikipedia pages on VEGF (note: Wikipedia is a free online encyclopedia that anyone can edit; available in several languages)

## Introduction

The classical role of VEGF in tumor progression is as a positive regulator of angiogenesis, the process of forming new capillaries from preexisting blood vessels [1]. Tumor growth is highly dependent on the ability of tumors to induce their own vascularization [2]. VEGF expression has been reported in a number of cancer cell lines and in several clinical specimens derived from breast, brain, and ovarian cancers [3–6]. Thus, antagonism of VEGF can effectively prevent tumor growth through incomplete blood vessel formation [7].

VEGF exerts its effects on endothelial cells in a paracrine mode after its release by other cells such as tumor cells, or in an autocrine manner in VEGF-producing endothelial cells. VEGF binds to its cognate receptors VEGFR1 (also known as FLT1), VEGFR2 (also known as KDR or FLK1), and neuropilin 1 (NRP1) [8]. VEGFR2 is responsible for initiating signal transduction pathways within endothelial cells. Following the binding of VEGF to VEGFR2, VEGF mediates its effects on proliferation, survival, adhesion, migration, capillary morphogenesis, and gene expression in endothelial cells [8]. VEGFR1 has a relatively minor role in VEGF-mediated signal transduction as compared to VEGFR2, since its kinase activity is 10-fold less than that of VEGFR2 [9]. Nonetheless, it has been shown that VEGFR1 induces growth factors from liver sinusoidal endothelial cells [10] and mediates the survival of endothelial cells through VEGF stimulation of the phosphatidylinositol 3-kinase/protein kinase B (AKT) pathway [2].

Breast cancer cell lines express both VEGF and the VEGF receptors VEGFR1, VEGFR2, and NRP1 [11]. The expression of these receptors in primary breast tumors is controversial. Some reports have documented the expression of VEGFR1 and VEGFR2 in breast tumors and others have reported very low to negative expression of both receptors. Furthermore, in one study the presence of VEGFR1 in breast cancer cells was shown to be significantly correlated with high metastasis risk and relapse and was considered a marker for breast tumor aggressiveness [12], while other studies did not detect the expression of VEGFR1 in breast tumors [13]. Thus, there are conflicting reports regarding the expression and role of VEGFR1 in breast cancer. Of note, no correlation of survival or metastasis risk and relapse was found in breast cancer cells that expressed VEGFR2 [12].

Recent studies have shown that VEGF acts as an autocrine growth and survival factor for VEGF receptor-expressing tumor cells [14–16]. However, the mechanism by which VEGF mediates the survival of tumor cells needs to be investigated in depth. Here, to address this issue, we analyzed the expression and function of VEGF and VEGF receptors in breast cancer cells.

## Methods

### Reagents

Human recombinant VEGF_165_ and anti-human VEGF monoclonal antibody were obtained from Genentech (http://www.gene.com). For all experiments, we used the VEGF_165_ isoform when VEGF is indicated. Anti-human VEGFR1 antibody recognizing the intracellular domain of VEGFR1 was obtained from Abcam (http://www.abcam.com; #Ab2350). Anti-human VEGFR1 antibody raised against the extracellular domain of VEGFR1 was obtained from R&D Systems (http://www.rndsystems.com; #MAB321), and from Santa Cruz Biotechnology (http://www.scbt.com). Anti-human VEGFR2 polyclonal antibody and PGF were purchased from R&D Systems. Anti-human NRP1 and anti-C-terminal Src kinase (Csk) antibodies were from Santa Cruz Biotechnology. VEGFR1 blocking antibody was a kind gift of Dr. Masabumi Shibuya (National University of Tokyo, Institute of Medical Science, Japan). This monoclonal antibody was shown to act as a functional blocking antibody against the VEGFR1 receptor [17].

### Cell Culture

The MDA-MB-231 and MCF-7 breast cancer cell lines were obtained from ATCC (http://www.atcc.org) and maintained in culture medium (DMEM or RPMI containing 10% FBS, 2 mM L-glutamine). Human brain microvascular endothelial cells (HBMECs) were purchased from Cell Systems (http://www.cell-systems.com) and maintained according to the methods described previously [18,19]. Human umbilical vein endothelial cells (HUVECs) were purchased from Cambrex BioScience (http://www.cambrex.com) and cultured with EGM-2 medium.

### Immunoprecipitation and Western Blot Analyses

Cells were lysed in kinase lysis buffer (New England Biolabs, http://www.neb.com) and lysates were incubated with primary antibodies at 4 °C. After overnight incubation, Protein G-Sepharose was added to the reaction mixture and incubated for 6 h. The immune complexes were precipitated with the Sepharose beads, washed, and resuspended in SDS-PAGE sample buffer with reducing agent. The samples were separated by 7.5% SDS-PAGE and transferred onto nitrocellulose membrane (Millipore, http://www.millipore.com). The membranes were blocked with 5% nonfat dried milk in PBS and subsequently incubated with primary antibodies overnight at 4 °C. Bound antibodies were detected by horseradish peroxidase-conjugated secondary antibodies and enhanced chemiluminescence (Amersham Pharmacia Biotech, http://www.amershambiosciences.com).

### Generation of MDA-MB-231 Cells Stably Transfected with Antisense VEGF

Human cDNA clones encoding VEGF were amplified by reverse transcription (RT) PCR using the following primers 5′-ACGACAGAAGGGGAGCAGAAAG-3′ (forward) and 5′-GGAACGTTGCGCTCAGACACA-3′ (reverse). These sequences were analyzed by the BLAST program (National Center for Biotechnology Information, http://www.ncbi.nlm.nih.gov/BLAST/) to confirm the specificity of these sequences and their absence from other genes. Subsequently, the 576 bp *VEGF* cDNA was cloned using the pBluescript II SK (+/−) vector (Stratagene, http://www.stratagene.com) and sequenced using the T7 promoter and primers. Both sense and antisense orientations were cloned into the Kpn1 restriction site of the constitutive mammalian expression vector, pZeoSV (Invitrogen, http://www.invitrogen.com), and sequenced using the T3 and SP6 primers. MDA-MB-231 cells were transfected with antisense VEGF vector and selected in the presence of Zeocin (phleomycin D1; 1 mg/ml). VEGF expression in the MDA-MB-231 transfectants was analyzed by Western blotting using polyclonal anti-VEGF antibodies (Santa Cruz Biotechnology).

### Terminal Deoxynucleotidyltransferase-Mediated Deoxy-UTP Nick End Labeling Assay

Cells were grown on chamber slides and stained with the fluorescein in situ cell death detection kit (Boehringer Mannheim, http://www.roche-applied-science.com), which is based on the terminal deoxynucleotidyltransferase-mediated deoxy-UTP nick end labeling (TUNEL) method, according to the protocol of the manufacturer. After they were washed with PBS, the cells were mounted and then intracellular fluorescein-labeled fragmented DNA was detected by microscopic analysis.

### RT-PCR

Total cellular RNA was isolated with TRIZol reagent (Invitrogen). The primers for *VEGF, VEGFR1,* and *VEGFR2* were purchased from R&D Systems, and the RT-PCR was performed as described in the manufacturer's protocol (BD Biosciences Clontech, http://www.bdbiosciences.com/clontech.shtml). PCR amplification was performed using 30 cycles for *VEGF* and 40 cycles for *VEGFR1* and *VEGFR2*.

### Small Interfering RNA

The small interfering RNA (siRNA) oligonucleotides used in this study were purchased from Dharmacon (http://www.dharmacon.com). A thorough analysis by BLAST was performed on all siRNA sequences used in this study to demonstrate that these sequences have no homology to other genes. Transfection procedures were performed with Oligofectamine Reagent (Invitrogen) in MDA-MB-231 cells or with DharmaFECT-1 reagent (Dharmacon) in MCF-7 cells according to the manufacturers' protocols. Cells were grown subconfluently on six-well plates and transfected with 200 nM of siRNA. After 5 d of culture, cells were washed and analyzed further as specifically indicated.

### Retroviral Infection

The siRNA-expressing pSIREN-RetroQ retroviral vector was used per the manufacturer's protocol (BD Biosciences Clontech). The wild-type and mutant *VEGFR2* siRNA targeting sequences are described in the Results section. For retroviral production, 293 Phoenix retrovirus packaging cells were cotransfected with Gag-Pol, pVSVG, and pSIREN-RetroQ retroviral vectors by using FuGENE 6 reagent (http://www.roche-applied-science.com). After 2 d of transfection, the supernatants were harvested, centrifuged, and passed through disposable filters of pore size 0.4 μm. The viral supernatants were then added to the MDA-MB-231 cells at a ratio of 1:1 (volume/volume) in the presence of polybrene (8 μg/ml). After 3 d of infection, cells were treated with puromycin (5 μg/ml) for selection of the stable cells expressing siRNA.

### Cell Cycle Analysis

Cells were harvested, centrifuged and fixed with 70% cold ethanol for a minimum of 2 h. Ethanol-fixed cells were centrifuged and washed once with PBS. The cell pellet was suspended in 0.5 ml of propidium iodide (PI) staining solution (0.1% Triton X-100, 20 μg/ml of PI, and 0.2 mg/ml of RNase in PBS) and incubated for 15 min at 37 °C. Samples were analyzed by flow cytometry, and apoptosis was measured as the percentage of cells with a sub-G_0_/G_1_ DNA content in the PI intensity–area histogram plot.

### Flow Cytometry Analysis

Cells were detached by incubation with 50 mM EDTA in PBS, centrifuged, and resuspended in PBS containing 1% BSA. Alternatively, the detached cells were permeabilized with 90% cold methanol for 10 min and resuspended in PBS containing 1% BSA. The cells were incubated with VEGFR1-specific antibodies (R&D Systems; #MAB321) or control antibodies for 30 min at 4 °C. After washing with PBS containing 1% BSA, the cells were incubated with FITC-conjugated secondary antibodies for 30 min at 4 °C. After a second washing with PBS, the cells were analyzed by flow cytometry.

### Immunocytochemistry

Confocal immunofluorescence staining was performed on methanol-fixed MCF-7 and MDA-MB-231 cells. We used two types of VEGFR1 antibodies: (a) antibodies against the VEGFR1 extracellular domain (R&D Systems or Santa Cruz Biotechnology) that interact with both the transmembrane form of VEGFR1 (the 200 kDa protein) and the soluble form of VEGFR1 (the 100 kDa protein); and (b) antibodies against the intracellular domain of VEGFR1 (Abcam; #ab2350), which recognize only the transmembrane full-length form of VEGFR1 and not soluble VEGFR1. The cells were permeabilized with PBST (0.1% and 0.5% Triton X-100 in PBS) and blocked with 3% normal goat or donkey serum in 0.1% PBST, then incubated in 3% normal goat serum in 0.1% PBST with primary antibodies against lamin A/C (LMNA) (rabbit polyclonal antibody, 1:100, Santa Cruz Biotechnology; H-110), LMNA (mouse monoclonal antibody, 1:50, Santa Cruz Biotechnology; #sc-7292), VEGFR1 (goat polyclonal antibody, 1:50, R&D Systems; #MAB321), and VEGFR1 (rabbit polyclonal antibody, 1:50, Abcam; #ab2350) overnight at 4 °C. Cells were then treated with FITC and Texas Red-conjugated secondary antibody (1:250, Jackson ImmunoResearch) diluted in 3% normal goat or donkey serum in 0.1% PBST for 1 h at room temperature. Cells were visualized with the 100× oil-immersion objective of a Zeiss 510 laser scanning microscope.

### Immunohistochemistry

To examine the expression of VEGFR1 and its localization at the nuclear membrane in tumor and breast tissues, immunohistochemical staining was carried out using anti-VEGFR1 (R&D Systems; #MAB321) and anti-lamin antibodies. Lamins are known to be the main components of intermediate filaments in the nuclear membrane. Tissue sections of human normal breast and invasive ductal breast carcinoma of varying pathological stages were immunostained for VEGFR1. Both the primary breast tumors and normal breast tissues were obtained from CHTN Eastern Division (University of Pennsylvania, Pennsylvania, United States), and were approved as part of a protocol of the Institutional Review Board at Beth Israel Deaconess Medical Center. The staining was performed on 5 μm sections, placed on polylysine-coated slides. After deparaffinization in xylene and rehydration through graded alcohol, each section was treated with 0.03% hydrogen peroxide for 5 min and blocked with 3% normal goat or donkey serum in 0.1% PBST, then incubated in 3% normal goat serum in 0.1% PBST overnight with primary antibodies against LMNA (rabbit polyclonal antibody, 1:100, Santa Cruz Biotechnology; #H-110), LMNA (mouse monoclonal antibody, 1:50, Santa Cruz Biotechnology; #sc-7292), VEGFR1 (goat polyclonal antibody, 1:50, R&D Systems; #MAB321), and VEGFR1 (rabbit polyclonal antibody, 1:50, Abcam; #ab2350) at 4 °C in a closed chamber. Following washing, cells were treated with the FITC and Texas Red-conjugated secondary antibody (1:250, Jackson ImmunoResearch) diluted in 3% normal goat serum in 0.1% PBST for 1 h at room temperature. Cells were visualized with the 100× oil-immersion objective of a Zeiss 510 laser scanning microscope.

### Nuclear Extraction

Nuclear and cytoplasmic fractionations were performed per the manufacturer's instructions (Pierce Biotechnology, http://www.piercenet.com). Briefly, 1,000 μl of CER I buffer was added to 100 μl of 5 × 10^6^ cells. After 10 min of incubation on ice, 55 μl of CER II buffer was added. Following incubation on ice for 1 min, cell suspension was centrifuged for 5 min at maximum speed (~16,000 *g*). The supernatants were transferred to a prechilled tube and kept on ice or at −80 °C until use. The pellet was then resuspended with 500 μl of NER buffer and incubated on ice for 40 min. Following centrifugation at the maximum speed for 10 min, supernatants were transferred to prechilled tubes and stored on ice or at −80 °C until use. To analyze the expression of VEGFR1 in the nuclear and cytoplasmic fractions, 100 μg of these extracts were loaded onto 4%–12% gradient SDS-PAGE gels, and transferred to nitrocellulose membranes. The blots were incubated with anti-VEGFR1 (R&D Systems; #MAB321), anti-lamin B1 (a nuclear marker), anti-GAPDH (a cytoplasmic marker), or anti-actin antibodies.

### Tumor Formation in Nude Mice

Athymic nude mice were used to assess the effect of siVEGFR1 on tumor growth in vivo. MDA-MB-231 cells were transfected with control siRNA (siCTL) or siVEGFR1 for 2 d. Aliquots of these cells (5 × 10^6^) were then analyzed for the knockdown of VEGFR1 expression by Western blotting. Mice (five per group) were subcutaneously inoculated in the flank area with either 5 × 10^6^ of siVEGFR1- or siCTL-MDA-MB-231 cells or with untransfected MDA-MB-231 cells. Tumor formation in these mice was monitored daily. The experiments were terminated at day 15, since some of the tumors in the control group reached a size of more than 1.5–2 cm^3^ (and therefore, based on the guidelines of our animal facility, we had to terminate the experiments). Tumor sizes were measured and expressed in cm^3^.

### Statistical Analyses

Data are presented as the mean ± standard deviation (SD). The Student *t*-test and one-way ANOVA were used to assess the significance of independent experiments. The criterion *p* < 0.05 was used to determine statistical significance.

## Results

### VEGF Receptor Expression in Human Breast Cancer Cells

**Figure 1:**
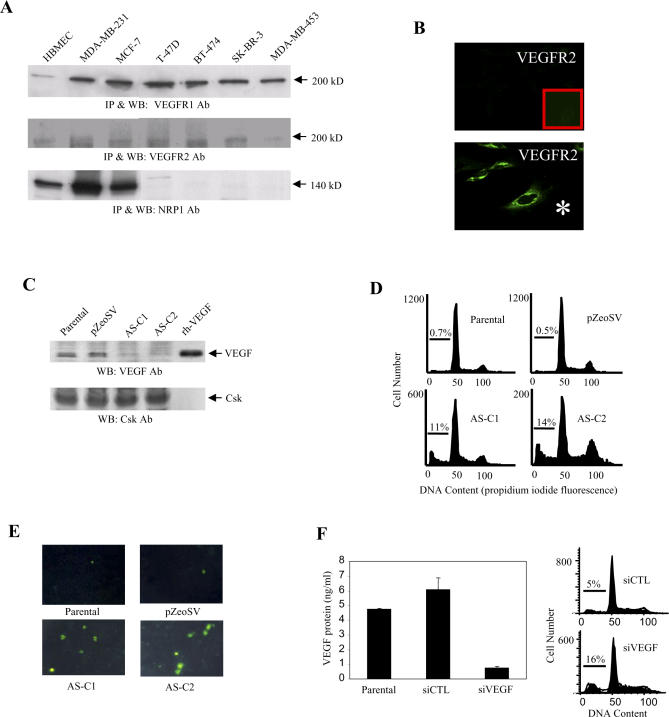
Expression of VEGF Receptors in Breast Cancer Cells and Apoptosis of MDA-MB-231 Cells by Down-Regulation of VEGF (A) VEGF receptor expression was examined by using immunoprecipitation (IP) and Western blot (WB) analyses in several breast cancer cell lines. (B) VEGFR2 expression in MDA-MB-231 cells was analyzed by confocal microscopy. Cells were stained with anti-VEGFR2 antibody or IgG (inset). The asterisk indicates cells transduced with adenovirus encoding soluble human VEGFR2, which served as a positive control. (C) MDA-MB-231 cells were transfected with antisense VEGF vector and selected in the presence of Zeocin (1 mg/ml). VEGF expression in the MDA-MB-231 transfectants (pZeoSV, AS-C1, and AS-C2) was assessed by Western blotting (WB) using a polyclonal anti-VEGF antibody. Total protein extracts were analyzed by Western blotting using anti-Csk antibody as an internal control. (D and E) MDA-MB-231 cells were stably transfected with antisense VEGF vector and the survival of these cells was measured by using cell cycle analysis (D) and TUNEL assay (E). (F) MDA-MB-231 cells were transiently transfected with siCTL or siVEGF oligonucleotides, and VEGF was quantitated in the supernatants (left graph) using an ELISA kit as directed by the manufacturer. The apoptosis of these cells was measured by using cell cycle analysis. The data are representative of three individual studies (right graphs).

Using immunoprecipitation and Western blot analyses, we examined expression of the VEGF receptors VEGFR1, VEGFR2, and NRP1 in several breast cancer cell lines. HBMECs were used as positive cells for VEGF receptor expression. The breast cancer cell lines include MDA-MB-231, MDA-MB-453, and BT-474, which are highly aggressive and form tumors and metastasis in vivo when inoculated into nude mice. The MCF-7 and T-47D breast cancer cell lines form tumors when inoculated into nude mice, but are less tumorigenic as they do not metastasize in vivo in the mice. We used 5 mg of total breast cancer cell lysates to examine the expression levels of VEGFR1, VEGFR2, and NRP1. VEGFR1 expression was abundant in all breast cancer cell lines, NRP1 expression was variable and observed primarily in MDA-MB-231 and MCF-7 cells, while the levels of VEGFR2 expression were low (Figure 1A). We next examined the expression of VEGFR2 in MDA-MB-231 cells by immunocytochemistry staining using anti-VEGFR2 antibody. For the positive control, MDA-MB-231 cells were transduced with adenovirus encoding soluble human VEGFR2. As shown in Figure 1B, unlike the MDA-MB-231 cells transduced with VEGFR2, no endogenous VEGFR2 expression was detected in untransfected MDA-MB-231 cells by immunocytochemistry analysis and confocal microscopy. These data suggest that VEGFR2 was expressed at relatively low levels compared to VEGFR1 and NRP1 in breast cancer cells. These results are consistent with our previous report detailing VEGFR2 mRNA expression in breast cancer cell lines [19].

As noted above, we found that both MDA-MB-231 and MCF-7 breast cancer cells expressed high levels of NRP1, while its expression was low in T-47D, BT-474, SK-BR-3, and MDA-MB-453 cells (Figure 1A). Thus, breast cancer cells express VEGF receptors in a differential manner, similar to the observations of VEGF receptor expression in breast tumor tissues [20]. Since MDA-MB-231 and MCF-7 cells express VEGFR1, VEGFR2, and NRP1 (Figure 1), and since MDA-MB-231 cells form tumors and metastasis in vivo in nude mice, while MCF-7 cells form tumors in nude mice but do not metastasize in vivo, both MDA-MB-231 and MCF-7 cell lines were chosen for further analysis, as described below.

### Down-Regulation of VEGF Expression Induces Apoptosis in MDA-MB-231 Cells

To examine the role of endogenous VEGF in breast cancer cells, we have generated MDA-MB-231 clones stably transfected with antisense *VEGF* cDNA constructs. As shown in Figure 1C, VEGF expression in these clones (AS-C1 and AS-C2) was dramatically down-regulated (about 70%, as determined by densitometry) compared to the control cells. The effects of *VEGF* antisense on apoptosis were also examined by cell cycle analysis and TUNEL assays. As shown in Figure 1D, cell cycle analysis revealed a higher percentage of apoptotic cells in the AS-C1 (11.28% ± 2.7%) and AS-C2 (14.47% ± 2.8%) clones, as compared to the parental (0.74% ± 0.05%) and pZeoSV control cells (0.51% ± 0.12%) (the results represent the mean ± standard deviation [SD] of three independent experiments). Similar apoptotic effects were observed in AS-C1 and AS-C2 cells using the TUNEL assay. Higher percentages of cells with apoptotic morphology were observed and quantitated in the AS-C1 (17.5% ± 0.7%) and AS-C2 (22.5% ± 2.12%) as compared to parental (2.5% ± 0.7%) and pZeoSV control (5.0% ± 2.8%) cells (these data reflect the mean ± SD of three independent experiments) (Figure 1E). To further confirm these results, MDA-MB-231 cells were transiently transfected with siRNA oligonucleotides for *VEGF* or with siCTL for 5 d. As shown in Figure 1F, siVEGF induced a knock-down in VEGF production and reduced the survival of the MDA-MB-231 cells as compared to the siCTL. Cells transfected with siCTL showed a low rate of apoptosis (5.07% ± 1.79%), whereas a 3-fold increase in apoptosis was observed in cells transfected with siVEGF (15.89% ± 1.91%). Thus, endogenous VEGF mediated the survival of MDA-MB-231 cells.

### Autophosphorylation of VEGFR2 in MDA-MB-231 Cells

**Figure 2:**
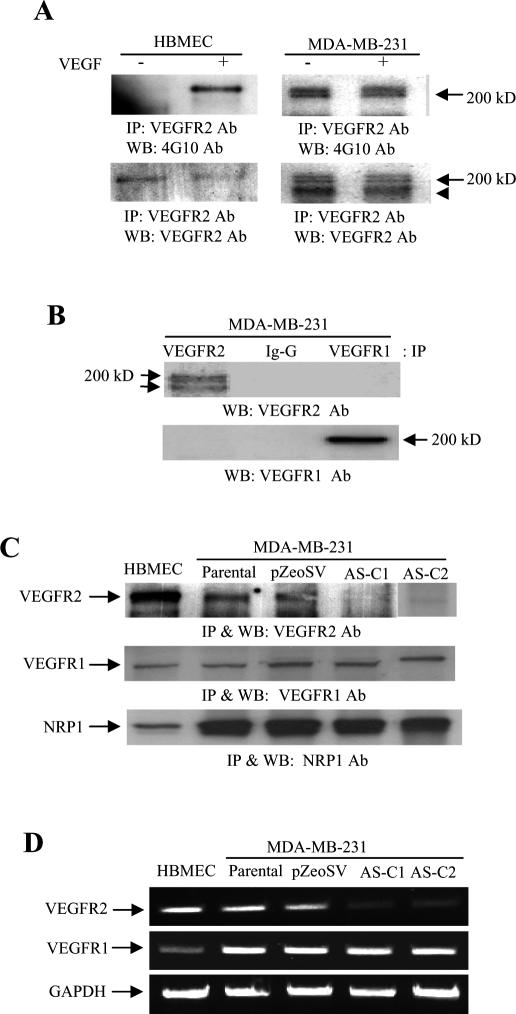
VEGF Receptor Expression in VEGF-Reduced MDA-MB-231 Cells (A) MDA-MB-231 cells and HBMECs were treated with VEGF (30 ng/ml) to examine its effects on the phosphorylation (top blots) and expression (bottom blots) of VEGFR2. Total cell lysates were prepared and then subjected to immunoprecipitation (IP) and Western blotting (WB) using 4G10 or anti-VEGFR2 antibodies. The arrowhead indicates a nonspecific protein band. (B) Lysates (5 mg) from MDA-MB-231 cells were immunoprecipitated by using anti-VEGFR1 or anti-VEGFR2 antibodies. The immune complexes were analyzed by Western blotting using anti-VEGFR2 antibodies. After stripping, the membrane was subjected to Western blotting using anti- VEGFR1 antibodies. (C) Lysates (2 mg) from variously transfected MDA-MB-231 cells were subjected to immunoprecipitation (IP) and Western blot (WB) analyses for VEGF and NRP1 receptor expression, as indicated. Total protein lysates (0.4 mg) from HBMECs were used as a positive control. (D) Total RNA (5 μg) from variously transfected MDA-MB-231 cells was subjected to RT-PCR analysis for *VEGFR1* or *VEGFR2* mRNA expression. *GAPDH* mRNA is shown as an internal control.

Next, we studied whether VEGF can induce the phosphorylation of VEGFR2 in MDA-MB-231 cells, similar to its effects in endothelial cells. HBMECs and MDA-MB-231 cells were treated with VEGF (30 ng/ml). Total cell lysates were prepared and immunoprecipitated with anti-VEGFR2 antibody and probed with 4G10 or anti-VEGFR2 antibodies. As shown in Figure 2A, the VEGFR2 receptor was phosphorylated in HBMECs upon VEGF stimulation, whereas the receptor was constitutively phosphorylated in MDA-MB-231 cells irrespective of VEGF stimulation. These data suggest that endogenous VEGF may be enough to induce activation of VEGFR2 in these tumor cells. Because VEGF induces signal transduction in endothelial cells via the formation of homo- and heterodimers of the VEGF receptors VEGFR1 and VEGFR2 [21], we therefore examined which complex of the two receptors is dominant in breast cancer cells. As shown in Figure 2B, we observed that VEGFR2 did not interact with VEGFR1, implying that endogenous VEGF may induce only the homodimer form of VEGFR2.

### Effect of VEGF on the Expression of VEGF Receptors

Based on the above experiments, we hypothesized that the decrease in VEGFR2 phosphorylation induced by the lower VEGF expression levels could cause the increased apoptosis of AS-C1 and AS-C2 clones. To test this possibility, AS-C1 and AS-C2 cell lysates (2 mg) were immunoprecipitated with VEGFR2 polyclonal antibodies, as indicated. Unexpectedly, we found that VEGFR2 protein expression was substantially decreased in the AS-C1 and AS-C2 cells as compared to that in the parental or pZeoSV cells (Figure 2C). We further confirmed these results using RT-PCR analysis (Figure 2D). The expression of VEGFR1 and NRP1 was not altered in the AS-C1 and AS-C2 cells (Figure 2C and 2D).

### Neither VEGFR2 nor NRP1 Mediates the Survival of MDA-MB-231 Cells

**Figure 3:**
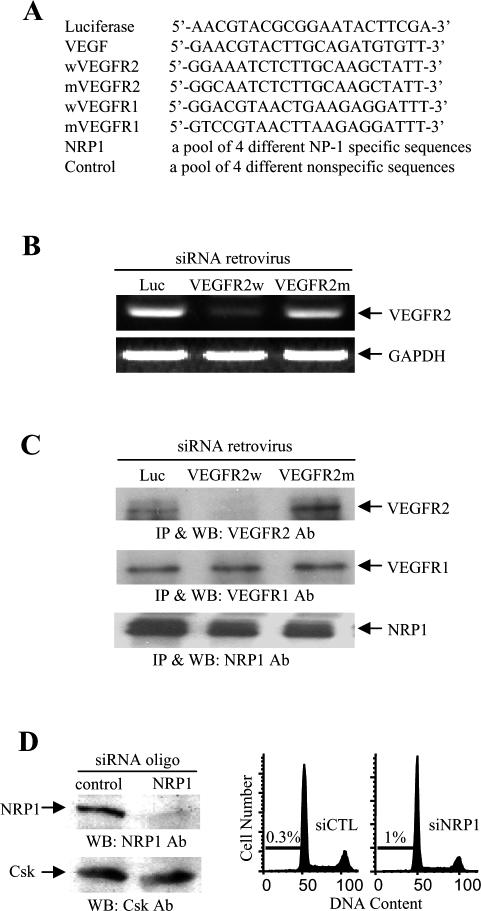
Effects of siVEGFR2 or siNRP1 Oligonucleotides on the Survival of MDA-MB-231 Cells (A) The siRNA oligonucleotides (and their sequences) used in this study were purchased from Dharmacon. (B) MDA-MB-231 cells were infected with retrovirus encoding wild-type (w) or mutant (m) siVEGFR2 or siLuc and then selected with puromycin (5 μg/ml). Total RNA (5 μg) was subjected to RT-PCR analysis for *VEGFR2* mRNA expression. *GAPDH* mRNA is shown as an internal control. (C) Total protein extracts (5 mg) from puromycin-selected cells were subjected to immunoprecipitation (IP) and Western blot (WB) analyses for VEGF and NRP1 receptor expression, as indicated. (D) MDA-MB-231 cells were transiently transfected with siCTL or siNRP1 oligonucleotides. After 5 d of incubation, total cell lysates were prepared and subjected to Western blotting (WB) using anti-NRP1 antibodies (left blots). Total protein extracts were analyzed by Western blotting using anti-Csk antibody as an internal control. The survival of siNRP1-treated cells was measured by using cell cycle analysis. The data are representative of three individual studies (right graphs).

To directly examine the effects of reducing VEGFR2 expression on the survival of MDA-MB-231 cells, we specifically lowered VEGFR2 expression using siVEGFR2 oligonucleotides. First, we used oligonucleotides for four different target sequences and examined the effects of each siRNA on *VEGFR2* mRNA transcription in the MDA-MB-231 cells using RT-PCR (unpublished data). Following these experiments, we selected a specific target sequence yielding high reduction in *VEGFR2* mRNA transcription (Figure 3A). We then constructed a retroviral vector encoding this sequence with which to infect the MDA-MB-231 cells. As shown in Figure 3B and 3C, these wild-type cells exhibited reduced VEGFR2 expression as compared to the luciferase- or mutated VEGFR2-siRNA-expressing cells, but no changes in VEGFR1 or NRP1 expression. However, no significant cell death was observed in the MDA-MB-231 cells expressing low levels of VEGFR2, as compared to the control or parental cells (unpublished data).

Next, we examined the effect of NRP1 on the survival of MDA-MB-231 cells, since VEGF was reported to mediate the autocrine survival of these cells through NRP1 [14]. MDA-MB-231 cells were preincubated with *NRP1*-targeted siRNA (siNRP1) oligonucleotides (200 nM) or with siCTL for 5 d. As shown in Figure 3D, while siNRP1 resulted in a complete reduction in NRP1 expression in the MDA-MB-231 cells, no cell death was observed in the siNRP1-treated cells (1.16% ± 0.25%) as compared to the siCTL-treated cells (0.29% ± 0.1%).

### VEGFR1 Mediates the Survival of MDA-MB-231 Cells

**Figure 4:**
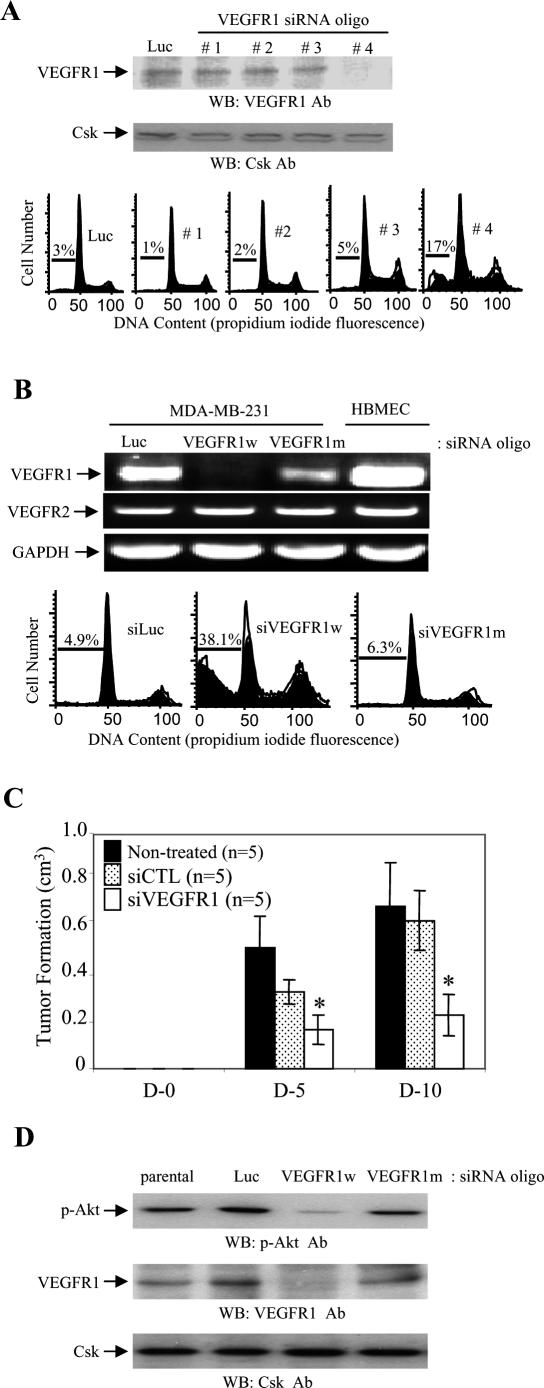
Effects of VEGFR1 siRNA Oligonucleotides on the Survival of MDA-MB-231 Cells (A) Effects of siVEGFR1 oligonucleotides on the apoptosis of MDA-MB-231 cells. MDA-MB-231 cells were transiently transfected with siLuc or with various siVEGFR1 oligonucleotides (#1 through #4). After 5 d of incubation, the cells were lysed and subjected to Western blotting (WB) using anti-VEGFR1 antibodies. Total protein extracts were analyzed by Western blotting using anti-Csk antibody as an internal control. The apoptosis of siVEGFR1-treated cells was analyzed by using cell cycle analysis, as indicated. (B) MDA-MB-231 cells were transiently transfected with wild-type (w) or mutated (m) siVEGFR1 oligonucleotides for 5 d. Total RNA (5 μg) was subjected to RT-PCR analysis for *VEGFR1* and *VEGFR2* mRNA expression; *GAPDH* mRNA is shown as an internal control (top gels). The survival of siVEGFR1-treated cells was measured by using cell cycle analysis (lower graphs). The data are representative of four individual studies. (C) MDA-MB-231 cells (5 × 10^6^), transfected with either siVEGFR1 or siCTL, were subcutaneously injected into the flank of athymic nude mice. Tumors were measured at days 0, 5, and 10 (D-0, D-5, D10, respectively) and expressed in cm^3^. Number of mice per group is indicated by *n*-value. *p < 0.05. (D) MDA-MB-231 cells were transiently transfected with siLuc or with wild-type (w) or mutated (m) siVEGFR1 oligonucleotides. After 5 d of incubation, total cell lysates were prepared and subjected to Western blotting (WB) by using phospho-AKT (p-AKT) or VEGFR1 antibodies. Total protein extracts were analyzed by Western blotting using anti-Csk antibody as an internal control for loading.

Since we noticed high expression of VEGFR1 in various breast cancer cell lines, we hypothesized that VEGFR1 may mediate VEGF-induced survival in breast cancer cells. To examine this possibility, we used oligonucleotides for four different target sequences of *VEGFR1* mRNA and examined the effects of each siRNA on VEGFR1 protein expression in the MDA-MB-231 cells upon Western blot analysis. We could clearly detect a VEGFR1 protein band corresponding to 200 kDa upon Western blotting with 0.1 mg of total cell lysates (Figure 4A). From these experiments, we found that one siRNA oligonucleotide (#4) among the four different siVEGFR1 oligonucleotides induced significant knockdown of VEGFR1 protein expression (Figure 4A). We also performed cell cycle analysis to determine the effects of siVEGFR1 on apoptosis. When MDA-MB-231 cells were treated with *luciferase*-targeted siRNA (siLuc), siVEGFR1#1, siVEGFR1#2, siVEGFR1#3 and siVEGFR1#4, the apoptosis rates were 3% (2.97% ± 0.26%), 1% (1.0% ± 0.15%), 2% (2.07% ± 0.16%), 5% (4.82% ± 0.42%), and 17% (17.04% ± 0.53%), respectively (Figure 4A). These data show that cells treated with siVEGFR1#4 resulted in a dramatic reduction in VEGFR1 expression and in a higher rate of apoptosis. To eliminate the nonspecific effect of functional siVEGFR1 on MDA-MB-231 cell survival, we used mutated siVEGFR1 oligonucleotides. The MDA-MB-231 cells were preincubated with wild-type or mutated siVEGFR1 oligonucleotides (200 nM) for 5 d. As shown in Figure 4B, the wild-type siVEGFR1-treated cells exhibited a reduction in *VEGFR1* mRNA transcription, as compared to the luciferase or mutant siVEGFR1-treated cells. Furthermore, these effects were specific for VEGFR1, since no changes were observed in VEGFR2 expression under these conditions (Figure 4B). Using cell cycle analysis, the apoptosis rates were 38.1% (38.14% ± 2.06%) in the MDA-MB-231 cells treated with wild-type siVEGFR1, as compared to cells treated with either luciferase (4.87% ± 0.45%) or mutant siVEGFR1 (6.28% ± 0.62%) (Figure 4B).

Next, we investigated the effects of siVEGFR1#4 on tumor formation in vivo in nude mice. MDA-MB-231 cells were treated with 200 nM of siVEGFR1#4 or siCTL for 2 d. Aliquots of these cells were then analyzed by Western blotting to verify the significant knockdown (>90%) of VEGFR1 in the siVEGFR1-treated cells. Cells were next harvested and inoculated into nude mice, and tumor formation was monitored in these mice. As shown in Figure 4C, the tumors in mice treated with siVEGFR1 were significantly smaller than those in mice treated with siCTL or in the untreated group. Thus, these in vitro and in vivo results demonstrate that VEGFR1 mediated the survival of the MDA-MB-231 cells.

### VEGFR1 Mediates AKT Phosphorylation in MDA-MB-231 Cells

It has been reported that VEGFR1 mediates the survival of endothelial cells through the phosphatidylinositol 3-kinase/AKT pathway [2]. Thus, to test whether VEGFR1 can also mediate breast cancer cell survival through this pathway, MDA-MB-231 cells were treated with siVEGFR1 oligonucleotides, and AKT phosphorylation was examined in these cells. As shown in Figure 4D, wild-type siVEGFR1 reduced AKT phosphorylation in MDA-MB-231 cells, whereas the luciferase-expressing cells or cells transfected with mutant siVEGFR1 had no effect. These data suggest that VEGFR1 might mediate the survival of MDA-MB-231 cells through AKT phosphorylation.

### The VEGF/VEGFR1 Axis Mediates the Survival of MCF-7 Breast Cancer Cells

**Figure 5:**
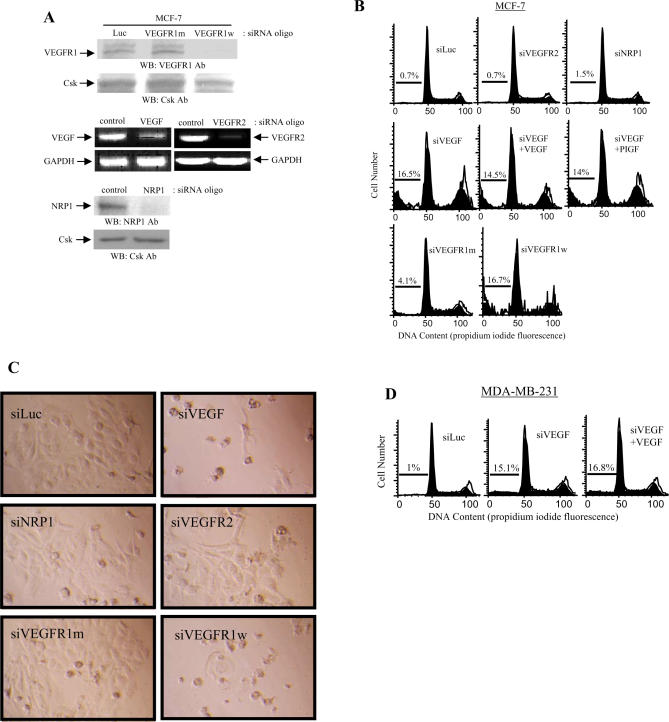
Effect of VEGF and VEGF Receptor siRNA Oligonucleotides on the Survival of MCF-7 Cells (A) MCF-7 cells were transiently transfected with various *VEGF* or *VEGF receptor* siRNA oligonucleotides, as indicated. Transfection procedures were performed with DharmaFECT-1 reagent according to the manufacturer's protocol. After 5 d of incubation, the cells were lysed and subjected to Western blotting (WB) using anti-VEGFR1 or anti-NRP1 antibodies. Total protein extracts were analyzed by Western blotting using anti-Csk antibody as an internal control. Alternatively, after 5 d of transfection, total RNA (5 μg) was isolated and subjected to RT-PCR analysis for *VEGF* and *VEGFR2* mRNA expression. *GAPDH* mRNA is shown as an internal control. (B) MCF-7 cells were transiently transfected with siLuc, siNRP1, siVEGF, or various *VEGF receptor* siRNA oligonucleotides in the absence or presence of PGF (20 ng/ml) or VEGF (20 ng/ml), as indicated. After 5 d of incubation, the cells were harvested and subjected to cell cycle analysis. The data are representative of three individual studies. m, mutant; w, wild type. (C) MCF-7 cells were transiently transfected with siLuc, siNRP1, siVEGF, or various *VEGF receptor* siRNA oligonucleotides as shown. After 5 d of incubation, the cells were observed under a light microscope. (D) Effect of VEGF on the cell cycle in MDA-MB-231 cells. MDA-MB-231 cells were transiently transfected with siLuc or with siVEGF oligonucleotides in the presence or absence of VEGF (20 ng/ml). After 5 d of incubation, cells were harvested and subjected to cell cycle analysis. The data are representative of three individual studies.

We next examined the effects of the VEGF/VEGFR1 axis on another breast cancer cell system, MCF-7 cells, in addition to MDA-MB-231 cells. As expected, the reduction in VEGF or VEGFR1 expression induced significant cell death in the MCF-7 cells, whereas the reduction in NRP1 or VEGFR2 expression had no significant effects on the survival of these cells (Figure 5A–5C). MCF-7 cells treated with siLuc, siVEGFR2, siNRP1, and mutant siVEGFR1 showed apoptosis rates of 0.7% (0.71% ± 0.09%), 0.70% (0.69% ± 0.19%), 1.50% (1.53% ± 0.30%) and 4.1% (4.2% ± 0.39%), respectively, while cells treated with either siVEGF (16.5% ± 0.69%), siVEGF with VEGF (14.54% ± 0.57%), siVEGF with PGF (13.95% ± 0.07%), or wild-type siVEGFR1 (16.70% ± 0.29%) showed a substantial increase in apoptosis. Thus, these data suggest that VEGF might mediate survival in a broad range of breast cancer cells via VEGFR1.

### Neither Exogenous VEGF nor Exogenous PGF Inhibits Apoptosis Following Reduced VEGF Expression in Breast Cancer Cells

**Figure 6:**
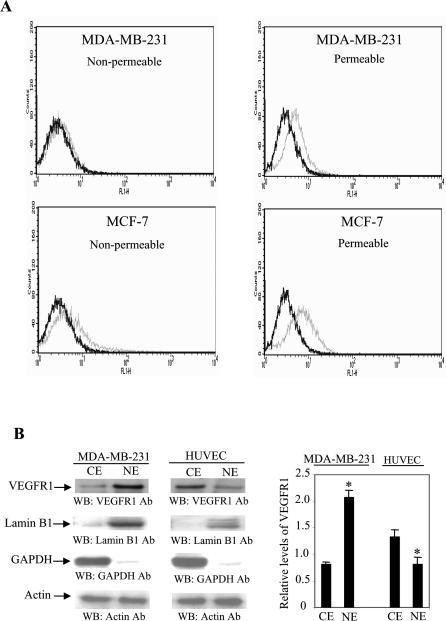
Internal Expression of VEGFR1 in Breast Cancer Cells (A) VEGFR1 expression in MDA-MB-231 and MCF-7 cells was analyzed using flow cytometry, under permeable and nonpermeable conditions. VEGFR1 expression (grey tracing) is detected mostly internally under permeable conditions. Staining with control antibody is indicated by the black tracing. (B) Cytoplasmic and nuclear fractions extracted from MDA-MB-231 cells and HUVEC were subjected to Western blotting (WB) using anti-VEGFR1, anti-lamin B1, anti-GAPDH, and anti-actin antibodies. The figure shows the relative expression levels of VEGFR1 in the cytoplasmic (CE) and nuclear (NE) extracts from MDA-MB-231 cells and HUVEC. *p < 0.05.

To further investigate whether VEGFR1 can play a role in the VEGF-mediated autocrine survival of MDA-MB-231 or MCF-7 cells, these cells were transiently transfected with siVEGF oligonucleotides in the presence of exogenous VEGF or the VEGFR1-specific ligand PGF (20 ng/ml). siVEGF resulted in a significant decrease in the survival of both MCF-7 and MDA-MB-231 cells (Figure 5B and 5D). Cell cycle analysis showed that the apoptosis rate of MDA-MB-231 cells treated with siVEGF was 15% (15.1% ± 0.23), which was about 15-fold higher than cells treated with siLuc (1.0% ± 0.26%) (Figure 5D). Furthermore, exogenous addition of VEGF or PGF to both MCF-7 and MDA-MB-231 cells did not abrogate the effects of siVEGF on the apoptosis of these cells (Figure 5B and 5D). Since PGF failed to rescue the survival of siVEGF-treated cells, this prompted us to study the localization of VEGFR1 expression in MDA-MB-231 and MCF-7 breast cancer cells. As shown in Figure 6, no VEGFR1 expression was detected at the surface of the MDA-MB-231 cells, while expression of VEGFR1 in the MCF-7 cells was low. The VEGFR1 antibodies used in these studies were specific for the detection of the full-length membrane form of VEGFR1. However, when these cells were permeabilized, we observed positive expression of VEGFR1 in both cell lines, indicating that VEGFR1 is expressed internally in both MDA-MB-231 and MCF-7 cells. We next examined the expression of VEGFR1 in the cytoplasmic and nuclear fractions of MDA-MB-231 cells as compared to HUVECs. Cellular fractions were subjected to Western blot analysis for VEGFR1, lamin B1 (a nuclear marker), GAPDH (a cytoplasmic marker), and actin. The levels of VEGFR1 expression in the cytoplasmic and nuclear fractions were quantitated by Scion image software. The data were normalized to the actin levels, and were expressed as relative units of VEGFR1 expression. The expression levels of VEGFR1 differed between the MDA-MB-231 cells and HUVECs (Figure 6B): a significantly higher level of VEGFR1 was detected in the nuclear fraction in MDA-MB-231 cells than in HUVECs. These results support the observation that VEGFR1 is mostly expressed internally in breast cancer cells.

**Figure 7:**
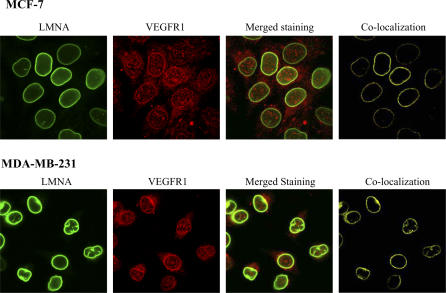
Localization of VEGFR1 in MCF-7 and MDA-MB-231 Cells Cells were stained with anti-VEGFR1 and anti-LMNA (Lamin A/C; a nuclear envelope marker) antibodies. Localization of VEGFR1 was then examined using confocal microscopy. LMNA was specifically localized at the nuclear envelope. Strong VEGFR1 protein expression was observed in the nuclear envelopes of the MCF-7 and MDA-MB-231 cells. Merged staining shows colocalization of VEGFR1 and LMNA at the nuclear envelopes.

**Figure 8:**
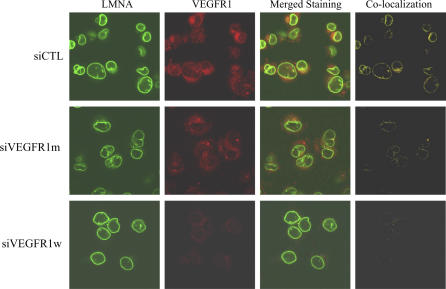
Localization of VEGFR1 in MDA-MB-231 Cells after siVEGFR1 Treatment MDA-MB-231 cells were transfected with siCTL, mutant siVEGFR1 (siVEGFR1m), or wild-type siVEGFR1 (siVEGFR1w), and then stained with anti-VEGFR1 and anti-LMNA (Lamin A/C) antibodies. Strong nuclear envelope staining and weak nuclear and cytoplasmic staining were observed for the VEGFR1 protein in the control siCTL and siVEGFR1m-transfected cells, whereas VEGFR1 protein expression was significantly decreased in the siVEGFR1w-transfected cells, and no VEGFR1 expression was observed in the nuclear envelope. The merged staining indicates the colocalization of VEGFR1 and LMNA.

To further determine the sublocalization of VEGFR1 in MCF-7 and MDA-MB-231 cells, we performed confocal analysis of these cells following their immunostaining with antibodies against the intermediate filament protein LMNA (which is expressed specifically at the nuclear envelope) and with VEGFR1 antibodies (which are specific to the extracellular domain of VEGFR1). As shown in Figure 7, VEGFR1 was mainly observed in the nuclear envelope of these cells. In addition, VEGFR1 was colocalized with LMNA at the nuclear envelope of both the MCF-7 and MDA-MB-231 cells. Merging of the VEGFR1 and LMNA immunostaining confirmed the colocalization of VEGFR1 with LMNA at the nuclear envelope in these cells. We further confirmed these results with additional antibodies against VEGFR1 that specifically recognize the membrane form of VEGFR1 and do not cross-react with soluble VEGFR1 (unpublished data). To demonstrate the specificity of the staining, we treated MDA-MB-231 cells with siCTL or with wild-type or mutant siVEGFR1, and analyzed the cells for the expression and sublocalization of VEGFR1. As shown in Figure 8, the siVEGFR1 treatments resulted in a substantial decrease in VEGFR1 expression in these cells. Specifically, no expression of VEGFR1 was observed in the nuclear envelope nor was any colocalization of VEGFR1 with LMNA found in these cells, as compared to the control siRNA-treated cells.

**Figure 9:**
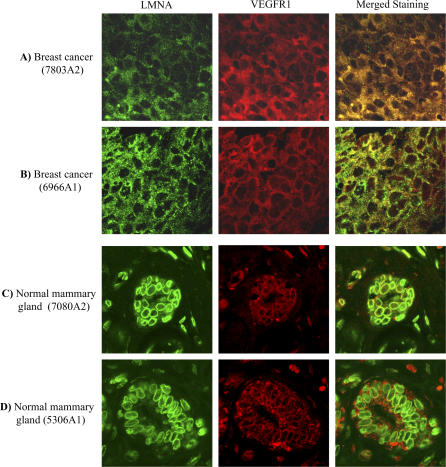
Localization of VEGFR1 Protein in Human Breast Tissues Tissue sections from human breast tumors and normal mammary glands were stained with anti-VEGFR1 and anti-LMNA (Lamin A/C) antibodies to determine their localization. (A and B) VEGFR1 was intensively colocalized with the nuclear envelope marker LMNA at the nuclear envelope and in the cytoplasm in human breast tumors. (C and D) VEGFR1 was colocalized with LMNA at the nuclear envelope in normal mammary glands.

We next examined the expression of VEGFR1 in ten sporadic breast cancer tumors. As shown in Figure 9, strong expression of VEGFR1 was found in the nuclear envelope and the cytoplasm of these tissues (Figure 9A and 9B), whereas VEGFR1 expression was mainly observed at the nuclear envelope in normal mammary gland tissues (Figure 9C and 9D). No membrane staining of VEGFR1 was observed in breast tumor or normal mammary gland tissues. Therefore, although VEGFR1 is expressed as a plasma membrane protein in endothelial cells, it is mainly expressed in the nuclear envelope in breast cancer cell lines, primary breast tumors, and normal mammary glands.

### VEGF and Its Protein Inhibitors Have No Biological Effects on Breast Cancer Cells

**Figure 10:**
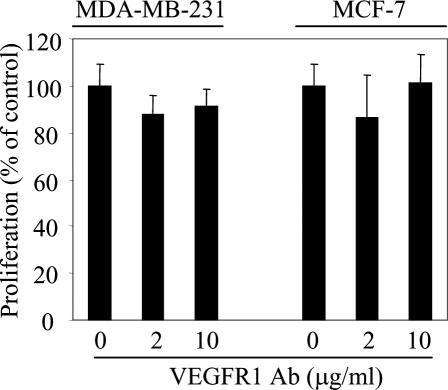
Effects of VEGFR1 Antibody on Cell Growth MDA-MB-231 and MCF-7 cells were grown subconfluently on six-well plates and treated with various concentrations of monoclonal antibody to VEGFR1. After 4 d of incubation, cells were harvested and counted on a hemocytometer. Data are presented as the means ± SD of three individual studies.

Based on the VEGFR1 localization results (Figure 6), we tested whether functional blocking of VEGFR1 would inhibit the growth or survival of breast cancer cells. For these experiments, we used VEGFR1-specific blocking antibody, which is known to inhibit the VEGF-induced migration of monocytes expressing VEGFR1 only [17]. MDA-MB-231 and MCF-7 cells were seeded onto six-well plates at a density of 5,000 cells per well. Following 4 d of treatment with the specific blocking antibody, cell proliferation was analyzed. The proliferation rates were similar in the MDA-MB-231 cells treated with 0, 2, and 10 μg/ml of VEGFR1 blocking antibody 100% (100% ± 9.16%), 88% (87.66% ± 8.08%), and 91% (91.11% ± 7.56%), respectively (Figure 10). Similar observations were made in MCF-7 cells treated with 0, 2, and 10 μg/ml of the VEGFR1 blocking antibody: the proliferation rates were 100% (100.12% ± 9.53%), 87% (86.66% ± 17.67%) and 100% (100.33% ± 12.70%) in the cells, respectively (Figure 10). Thus, this antibody did not inhibit the proliferation of the MDA-MB-231 and MCF-7 cells. Using cell cycle analysis, we confirmed that this antibody had no effect on the survival of these cancer cells, since VEGFR1 is mostly expressed internally and not on the surface of breast cancer cells as expected (unpublished data). These results are consistent with our previous report showing that VEGF had no effect on the invasion of MDA-MB-231 cells [19]. In addition, VEGF blocking antibody or soluble VEGFR1 protein had no effect on the survival or proliferation of MDA-MB-231 cells (unpublished data). Taken together, these data indicate that VEGF plays a role as an intracrine survival factor in breast carcinoma cells through VEGFR1, which is mainly expressed internally.

## Discussion

In this study, we found that several breast cancer cell lines expressed VEGF receptors. Furthermore, we showed that reduced endogenous VEGF or VEGFR1 expression induced the apoptosis of MDA-MB-231 and MCF-7 breast cancer cells, whereas externally acting proteins (VEGF, VEGF antibody, soluble VEGFR1, and VEGFR1-blocking antibody) had no significant effects on breast cancer cell growth or survival. We also observed that internally expressed VEGFR1 induced the survival of breast cancer cells. These results indicate that VEGF plays a role as an internal autocrine survival factor in breast cancer cells via VEGFR1 and not through VEGFR2 or NRP1, and suggest that the role of the VEGF/VEGFR1 axis in breast cancer cells may not be governed by the classical paradigm of signal transduction involving interaction between ligands and cell surface receptors.

VEGF contributes to the growth of solid tumors, such as breast cancer, through angiogenesis rather than through a direct contribution to tumor cell survival. This effect seems to be derived partly from the poor response of tumor cells to externally acting molecules such as VEGF or antibodies to VEGF. However, this concept has now been challenged by recent reports showing that some cancer cells express VEGF receptors on their surfaces in vitro and in vivo, and that in several tumor cell types, including leukemia and melanoma cells, VEGF acts as an autocrine survival factor [22,23]. Although breast cancer cell lines express both VEGF and the VEGF receptors VEGFR1, VEGFR2, and NRP1 [11], the expression of these receptors in primary breast tumors is controversial [12,13,24–28]. Dales et al [12] reported that VEGFR1 and VEGFR2 were strongly expressed in endothelial cells within blood microvessels, and weakly expressed in tumor cells in a series of 918 patients. Univariate analysis showed that the expression of VEGFR1 in tumor cells was not correlated with survival, but was significantly correlated with high metastasis risk [12]. In another study involving 905 cases of breast cancer tumors [29], the expression of VEGFR1 was correlated with a high risk of local recurrence. On the other hand, VEGFR1 expression was rarely observed in 205 cases of ductal carcinoma in situ of the breast [13]. Thus, there are conflicting reports regarding the expression and role of VEGFR1 in breast cancer. No correlation between survival and metastasis risk and relapse was found in breast cancer cells that expressed VEGFR2 [12]. Therefore, the role of VEGF as an angiogenic factor and/or as a survival factor in tumor cells needs to be fully investigated.

Our siRNA studies on VEGF receptors revealed that VEGF can modulate the survival of breast cancer cells via VEGFR1, but not VEGFR2. Here, we show that VEGFR1 mediated AKT phosphorylation, a major survival pathway in breast cancer cells. It is known that VEGF mediates most angiogenic processes by interacting with VEGFR2 in endothelial cells. VEGF can also modulate VEGFR2 expression as well as its phosphorylation in endothelial cells [30]. Thus, down-regulation of VEGF expression may induce apoptosis in the MDA-MB-231 clones AS-C1 and AS-C2 through the decreased VEGFR2 expression in these cells. However, our studies with siVEGFR2 demonstrated that VEGFR2 is not important for the survival of breast cancer cells. Although VEGFR2 has strong tyrosine kinase activity compared to VEGFR1 in endothelial cells, the level of VEGFR2 expression in MDA-MB-231 and MCF-7 breast cancer cells might not be sufficient to induce VEGF-mediated signaling in these cells. In fact, we almost could not detect VEGFR2 protein expression by immunoprecipitation, nor could we detect any expression of this protein by immunocytochemical analysis (Figure 1A and 1B).

VEGFR2 tyrosine phosphorylation was reported to be enhanced by VEGF in breast cancer and to lead to increased ERK1/2 (extracellular signal-regulated kinase 1/2) and AKT phosphorylation, suggesting that VEGF stimulation is important in the regulation of cell growth, apoptosis, and differentiation [31]. VEGFR2 was also shown to be expressed on the surface of breast cancer cells [31]. However, these results differ from our data since the level of expression of VEGFR2 was very low in our cell system, and knockdown of VEGFR2 by siRNA treatment had no effect on the apoptosis of breast cancer cells. Additionally, we examined the effect of NRP1 on the survival of MDA-MB-231 cells, as it was previously reported that VEGF mediates the autocrine survival of these cells through NRP1 [14]; we failed to show that NRP1 mediated VEGF-induced survival in MDA-MB-231 cells. NRP1 is known to interact with VEGFR1 but has a very short cytoplasmic domain [32], suggesting that NRP1 may not directly transduce VEGF-mediated signaling in these cells. Although we can not fully explain these discrepancies, based on our results it is unlikely that VEGFR2 or NRP1 has a direct functional effect on VEGF-mediated survival in MDA-MB-231 cells. Knockdown of endogenous VEGF expression or addition of VEGFR synthetic inhibitors resulted in the apoptosis of hematopoietic stem cells [33], whereas VEGF or soluble VEGFR1 did not have any effect on the survival of these cells. Therefore, the survival mechanism of VEGF in breast cancer cells might be different from that of VEGF in endothelial cells, and may mimic the VEGF-dependent survival system in hematopoietic stem cells [33].

VEGFR1 was originally thought to be a receptor specifically expressed in vascular endothelial cells. However, some studies have shown that VEGFR1 is expressed in tumor cells and is involved in tumor growth and progression [5,6,20,34]. VEGF has also been shown to increase the invasiveness of colorectal cancer cells or myeloma cells [15,16], and VEGFR1-blocking antibody was reported to inhibit the VEGF-induced invasiveness of these cells [15,16]. These results indicate that VEGF can act as an autocrine or paracrine factor in the invasiveness of both cell types via VEGFR1. These previously published studies are different from our present data, which show that neither exogenous VEGF nor VEGFR1-blocking antibody has any significant effects on the invasiveness or survival of breast cancer cells. We observed that VEGFR1 was expressed internally, but not on the surface of breast cancer cells. The localization of VEGFR1 expression in breast cancer cells is a novel finding that may support our observations that VEGF plays a role as an internal autocrine survival factor in these cells. It remains to be elucidated how VEGFR1 is expressed internally in breast cancer cells, and how it mediates VEGF-induced survival in these cells.

The results of our studies differ from those of studies by Vincent et al. [16] showing that VEGF facilitated the growth and survival of human primary multiple myeloma cells via VEGFR1. In that study, VEGFR1 was shown to be present in the cytoplasm and the nuclei of proliferating multiple myeloma cells. Vincent et al. [16] reported that a monoclonal antibody against VEGFR1 inhibited the proliferation and migration of primary multiple myeloma cells via plasma membrane retention of VEGFR1 following the prevention of its nuclear translocation. In our study, however, externally acting growth factors, such as VEGF and VEGFR1-blocking antibody, had no effects on breast cancer cell growth or survival. Furthermore, VEGFR1 receptors were mostly expressed internally, and not on the surface of breast cancer cells, which may provide a mechanism for the lack of inhibition of breast cancer cell growth by antibody to VEGFR1. We also observed localization of VEGFR1 mainly in the nuclear envelope in breast cancer cell lines, primary breast cancer tumors, and normal mammary glands. These results suggest that the expression pattern, localization, and function of VEGFR1 may differ between breast epithelial cells and endothelial cells.

Although VEGF has a signal peptide that induces its direct secretion through the classical secretory pathway, VEGF can also translocate to the nucleus [35]. Moreover, VEGF contains basic amino acids that could act as potential nuclear localization signals [36]. Recent studies showed that VEGF protein colocalizes with the RNA-binding protein HuR in discrete nuclear compartments and that nuclear VEGF protein is increased in hypoxia [36]. These results indicate that VEGF may have a nuclear function, especially during hypoxia. Furthermore, VEGF nuclear accumulation is correlated with phenotypic changes in endothelial cells [30]. VEGF is internalized via the classical receptor-mediated endocytosis pathway and accumulates in the endosomal compartment, whereas in cells situated at the edges of a wound, VEGF is rapidly taken up and translocated to the nucleus [30]. Therefore, it is possible that VEGF may be retained within the cell for its interaction with perinuclear VEGFR1. Future studies will address the function of VEGFR1 in live breast cancer cells and normal breast epithelial cells, and determine the kinetics, translocation, and binding of VEGFR1 to VEGF in these cells.

The limitation of our study is that the intracrine role of VEGF/VEGFR1 was examined in two breast cancer cell lines and in only a few samples of primary breast tumors. These results may not be fully applicable to all breast cancer samples, representing different stages of the disease. Thus, further studies of breast cancer cells from patients at different clinical stages of the disease will be necessary to define the pathophysiologic role of the VEGF/VEGFR1 axis in intracrine signaling. In addition, although we were able to examine the functional blocking of VEGFR1 using antibodies that were readily accessible to our laboratory, other VEGFR1 antibodies (that are currently unavailable to us) need to be examined for their effects on breast cancer cell growth and proliferation in vivo in this system, once these antibodies become available.

Recently it was reported that VEGFR1 expression was significantly increased in breast cancer patients with a poor prognosis [37]. Consistent with that report, our data suggest that VEGFR1 might be an attractive target for therapeutic approaches in patients with malignant breast cancer. Furthermore, our data suggest that intracellularly acting inhibitors against VEGF and its receptor VEGFR1 might be a more effective therapeutic method for breast cancer patients as compared to the externally acting inhibitors. Specifically, the former approach will exert direct tumor-killing effects as well as antiangiogenic effects, while the latter approach will exert indirect tumor-killing effects through antiangiogenic activity alone. Hence, our study may provide an optimal strategy for tumor therapy based on the inhibition of angiogenesis.

## Supporting Information

### Accession Numbers

The GenBank (http://www.ncbi.nlm.nih.gov/) accession numbers of the genes/proteins discussed in this paper are *lamin A/C* (*LMNA;* NM_170707), *lamin B1* (*LMNB1;* NM_005573), *neuropilin 1* (*NRP1;* AF016050), *placental growth factor* (*PGF;* S72960), *vascular endothelial growth factor* (*VEGF;* AB021221), *vascular endothelial growth factor receptor 1* (*VEGFR1/FLT1;* AF063657), and *vascular endothelial growth factor receptor 2* (*VEGFR2/KDR/FLK1;* AF035121).

## Abbreviations

Csk: C-terminal Src kinase
HBMEC: human brain microvascular endothelial cell
HUVEC: human umbilical vein endothelial cell
LMNA: lamin A/C
NRP1: neuropilin 1
PGF: placenta growth factor
PI: propidium iodide
RT-PCR: reverse transcription PCR
SD: standard deviation
siCTL: control siRNA
siLuc: *luciferase*-targeted siRNA
siRNA: small interfering RNA
siNRP1: *NRP1*-targeted siRNA
siVEGF: *vascular endothelial growth factor*-targeted siRNA
siVEGFR: *vascular endothelial growth factor receptor*-targeted siRNA
VEGF: vascular endothelial growth factor
VEGFR: vascular endothelial growth factor receptor
